# 897. Trends and Correlation of HIV-1 Reservoir in Acute HIV Infection and Chronic HIV Infection in China

**DOI:** 10.1093/ofid/ofab466.1092

**Published:** 2021-12-04

**Authors:** Shuang peng, Ming wang

**Affiliations:** The First Hospital of Changsha, Changsha, Hunan, China

## Abstract

**Background:**

Among acute HIV infection （AHI）and chronic HIV infection（CHI）,the association of HIV-1 DNA and HIV-1 RNA is currently a hot spot of concern. We studied HIV-1 DNA levels in patients with AHI and CHI before initiation of ART to explore the growth characteristics of the HIV reservoir.

**Methods:**

From 2016/10/31 to 2020/11/23, 97 patients were enrolled in the first hospital of Changsha in China. According to the patient’s epidemiological history, HIV-1 antibody conversion time, presence of opportunistic infection（OI）, to determine whether the patients were in the acute or chronic infection period, and divided into two arms: AHI and CHI. Lleukomonocyte, HIV-1 RNA, and CD4/8 of all patients were collected. The HIV-1 DNA in peripheral blood mononuclear (PBMC) was detected by PCR-Fluorescence Probing. The results were analyzed by SPSS 22.0 and GraphPad Prism 8.0. P-value < 0.05 were statistically significant.

**Results:**

93 of 97 were male and 85 of 97 with sexual transmission. In AHI arm, the mean of HIV-1 RNA was 5.15 log10 copies/ml, and the mean of HIV-1 DNA was 2.83 log10 copies/10^6^ PBMCs. In CHI Arm, the mean value of HIV-1 RNA was 4.90 log10 copies/ml, and the mean value of HIV-1 DNA was 3.19 log copies/10^6^ PBMCs. The HIV-1 DNA of CHI group was higher than that of AHI group (p = 0.002) , but the HIV-1 RNA of CHI group was lower than that of AHI Group (p = 0.183) . There were no significant differences between AHI and CHI in age, sex, body weight, route of infection, ART, other viral infection, leukomonocyte, CD4+ T cell count, CD4+ T cell percentage, CD8+ T cell count, CD8+ T cell percentage and CD4/CD8 ratio (P > 0.05).In Group AHI, HIV-1 DNA was positively correlated with HIV-1 RNA (r = 0.548, p < 0.001), but not in Group CHI (r = 0.14, p = 0.347).

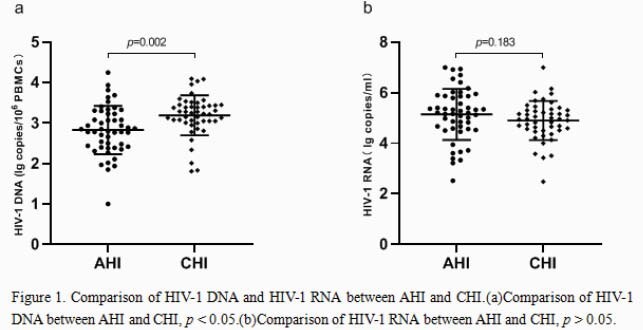

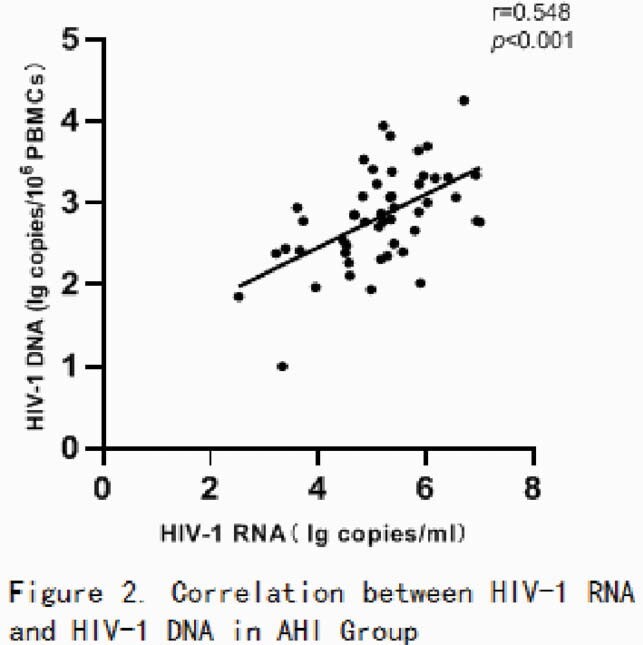

**Conclusion:**

Patients with AHI have lower HIV-1 DNA levels and smaller viral reservoir than those with CHI. These data have illustrates the benefits of rapid treatment. The correlation between HIV-1 DNA and HIV-1 RNA in patients with acute infection is strong,the level of HIV-1 DNA increased with the increase of HIV-1 RNA level, but was not related to CD4 + T cells, CD8 + T cells and CD4/CD8 ratio.

**Disclosures:**

**All Authors**: No reported disclosures

